# P-839. Not All Bugs Need Drugs: Leveraging ePlex Blood Culture Identification (BCID) for Smarter Stewardship

**DOI:** 10.1093/ofid/ofaf695.1047

**Published:** 2026-01-11

**Authors:** William Harris, David S Burgess, Vaneet Arora, Donna R Burgess, Ashley Logan, Ryan P Mynatt, Katie B Olney, Jeremy VanHoose, Katie L Wallace, Sarah Garvey

**Affiliations:** University of Kentucky HealthCare, Lexington, Kentucky; University of Kentucky, Lexington, KY; University of Kentucky, Lexington, KY; UK HealthCare, Lexington, KY; University of Kentucky HealthCare, Lexington, Kentucky; University of Kentucky, Lexington, KY; University of Kentucky HealthCare, Lexington, Kentucky; UK HealthCare, Lexington, KY; UK HealthCare, Lexington, KY; University of Kentucky HealthCare, Lexington, Kentucky

## Abstract

**Background:**

Bacillus, Micrococcus, Cutibacterium, Corynebacterium and Lactobacillus species identified in blood cultures are often considered contaminants and their treatment leads to unnecessary antimicrobial utilization, prolonged hospitalization, and increased healthcare costs. While blood culture identification (BCID) rapid diagnostic technologies combined with stewardship interventions demonstrate improved outcomes, limited data exist on their impact for contaminants beyond coagulase negative staphylococci. This study assessed the impact of ePlex® BCID on antimicrobial utilization for non-staphylococcal contaminated blood cultures.

**Methods:**

Patients with a positive blood culture for Bacillus cereus group, Bacillus subtilis group, Corynebacterium species (spp.), Cutibacterium acnes, Lactobacillus spp., and Micrococcus spp. between January 2017 and October 2023 were included. Blood culture results and antimicrobial data were evaluated before (relied on MALDI-TOF) and after the implementation of ePlex® BCID panels at UK HealthCare. The primary outcome was total days of targeted antimicrobial therapy per patient.

**Results:**

A total of 142 unique patients were included in the pre-ePlex® group (January 2017-June 2020) and 261 patients in the post-ePlex® group (June 2021-October 2023). The baseline patient demographics were well-balanced. Median length of antimicrobial therapy (4.5 vs 3.5 days, p = 0.60) and vancomycin therapy (2.0 vs 2.9 days, p = 0.54) was not different between groups; however, patients in the post group were more likely to be started on antibiotics within 24 hours of blood culture collection (40.1 vs 51.7%, p = 0.03) and to receive vancomycin during their hospital stay (49.3 vs 59.8%, p = 0.04). Time to organism identification was shorter in the post-group versus the pre-group (5.5 vs 2.3 days, p < 0.001). Infectious diseases consultation was significantly higher in the post-group (7.7 vs 29.1, p < 0.001).

**Conclusion:**

This study highlights antimicrobial stewardship opportunities associated with non-staphylococcal contaminants. Leveraging rapid diagnostic technology in conjunction with antimicrobial stewardship may optimize antimicrobial use and improve outcomes by facilitating earlier identification and targeted stewardship interventions.

**Disclosures:**

Sarah Garvey, PharmD, BCIDP, Gilead: Advisor/Consultant

